# Pathogenicity in chickens and ducks of clade 2.3.4.4b H5N6 and H5N3 high pathogenicity avian influenza viruses in South Korea

**DOI:** 10.3389/fvets.2026.1833958

**Published:** 2026-06-26

**Authors:** Yunyueng Jang, Ra Mi Cha, Min-Ji Park, Jinmyeung Kim, Jong-Min Kim, Eui Hyeon Lim, Youn-Jeong Lee, Eun-Kyoung Lee

**Affiliations:** Avian Influenza Research and Diagnostic Division, Animal and Plant Quarantine Agency, Gimcheon, Republic of Korea

**Keywords:** chicken, duck, H5N3, H5N6, high pathogenicity avian influenza, pathogenicity

## Abstract

High pathogenicity avian influenza viruses (HPAIVs) of the A/Goose/Guangdong/1/96 lineage continue to diversify through reassortment, generating various H5Nx subtypes. In South Korea, H5N6 HPAIVs were introduced in wild birds and poultry during the 2023–2024 winter season, and the novel reassortant H5N3 HPAIV was first identified in wild birds in late 2024. Herein, we evaluated the pathogenicity and transmissibility of two recently emerged clade 2.3.4.4b H5 HPAIV subtypes—A/duck/Korea/D449/2023(H5N6) [D449(H5N6)] and A/northern pintail/Korea/WF369-1/2024(H5N3) [WF369-1(H5N3)]—in specific pathogen free chickens and commercial ducks. In chickens, the mean death time of the D449(H5N6) and WF369-1(H5N3) groups was 2.6 and 2.2 days, respectively, and the median chicken lethal dose (cLD_50_) of both viruses was calculated to be 10^4.5^ median egg infectious dose (EID_50_). In contact chickens, D449(H5N6) achieved 100% transmission, whereas no transmission was observed in chickens exposed to WF369-1(H5N3), suggesting the rapid mortality (MDT 2.2 days) likely limited the window for horizontal transmission. Ducks inoculated with D449(H5N6) showed distinct clinical signs, including respiratory signs, which correlated with elevated viral titers in the lung and trachea. In contrast, ducks inoculated with WF369-1(H5N3) exhibited neurological signs, with viral replication in all internal organs. In addition, D449(H5N6) showed significantly higher infectivity with a median bird infectious dose (BID_50_) of <10^2^ EID_50_, while the BID_50_ for WF369-1(H5N3) was 10^5^ EID_50_. These strain-specific differences in virulence and transmissibility provide relevant information for early detection of viruses and disease control strategies in poultry. Our findings underscore the need for continued investigation to offer additional insight for field-level risk assessments.

## Introduction

The A/goose/Guangdong (gs/GD/96) lineage of H5 high pathogenicity avian influenza viruses (HPAIVs) has diversified into multiple phylogenetically distinct hemagglutinin clades (0–9). Since 2014, clade 2.3.4.4 has been pre-dominant globally, and its subclades (a–h) have caused widespread outbreaks (1). Notably, clade 2.3.4.4b spread rapidly following the emergence of H5N8 HPAIVs in wild birds and domestic poultry (2). Subsequently, clade 2.3.4.4b H5N1 caused the largest recorded HPAI epidemics during 2021–2022, undergoing extensive reassortment across various continents, including Europe, Asia, Africa, and North America (3, 4). Furthermore, multiple H5Nx subtypes, including H5N1, H5N2, H5N3, and H5N5, have emerged through reassortment with low pathogenicity avian influenza viruses (5–7).

Although H5N1 HPAIVs have pre-dominated globally in recent years, genetically diverse clade 2.3.4.4b H5Nx viruses continue to emerge in poultry and wild birds across East Asia. During the 2023–2024 winter season, H5N1 and H5N6 HPAIVs were introduced into East Asia by migratory birds (8, 9). The H5N6 HPIAVs detected in South Korea, Japan, and China were genetically similar, indicating regional circulation within East Asia (10). Subsequently, H5N5 HPAIVs with unique gene constellations were identified in Japan, likely introduced through stepwise dissemination along migratory bird flyways across northern Eurasia (11). At the beginning of the 2024–2025 winter season in South Korea, a novel clade 2.3.4.4b H5N3 HPAIV was first reported from a northern pintail. Phylogenetic analysis identified this virus as a novel reassortant with gene segments distinct from those of previously reported H5N3 HPAIVs (10). Although clade 2.3.4.4b H5N3 HPAIVs were rarely reported in Europe in 2021, their pathogenicity in domestic poultry remains uncharacterized.

The continued emergence of genetically diverse H5Nx HPAIVs is associated with a broad spectrum of virulence determinants that influence infection dynamics. H5 HPAIVs typically induce acute systemic infection and high mortality in chickens, facilitating rapid spread within flocks (12–15). In contrast, ducks exhibit variable clinical manifestations following H5 HPAI infection and may act as asymptomatic reservoirs, contributing to viral persistence and transmission among wild and domestic avian species (14, 16). Therefore, evaluating the virulence of newly emerging H5Nx HPAIVs is essential to assess their potential impact on poultry production and interspecies transmission. In this study, we investigated the pathogenicity and transmissibility of two recently emerged H5 HPAI subtypes in South Korea, H5N6 and H5N3, in chickens and ducks.

## Materials and methods

### Viruses

A/duck/Korea/D449/2023(H5N6) [D449(H5N6)], isolated from a broiler duck, and A/northern pintail/Korea/WF369-1/2024 (H5N3) [WF369-1(H5N3)], obtained from fecal samples of wild birds, were propagated in 10-day-old specific pathogen–free (SPF) embryonated chicken eggs (CE3) as challenge viruses for animal experiments (Supplementary Table S1). Eggs were incubated at 37 °C for up to 72 h, and those with dead embryos were immediately chilled at 4 °C. Hemagglutination-positive allantoic fluid was harvested and stored at −70 °C until further use.

### Experimental design

SPF chickens (5 weeks old, *n* = 82) and commercial ducks (2 weeks old, *n* = 52) were used. All birds were confirmed seronegative for avian influenza virus antibodies by competitive enzyme-linked immunosorbent assay (cELISA; BIONOTE). Birds were housed in negative-pressure isolators equipped with high-efficiency particulate air filtration under biosafety level 3 conditions. Feed and water were provided *ad libitum*.

In chickens, pathogenicity was assessed using the intravenous pathogenicity index (IVPI) according to WOAH guidelines. Ten SPF chickens per virus were inoculated intravenously with 0.1 ml of a 1:10 dilution of infectious allantoic fluid containing either D449(H5N6) or WF369-1(H5N3). The median chicken lethal dose (cLD_50_) was determined by intranasal inoculation of four groups (*n* = 5 per group) with 10-fold serial dilutions of each virus ranging from 10^6^ to 10^3^ median chicken lethal dose (EID_50_). In ducks, the median bird infectious dose (BID_50_) was estimated by intranasal inoculation of three groups (*n* = 5 per group) with 10^2^, 10^4^, or 10^6^ EID_50_/0.1 ml of either D449(H5N6) or WF369-1(H5N3). The cLD_50_and BID_50_ was calculated by Reed-Muench method. To evaluate transmissibility, three naïve contact chickens or ducks were co-housed with the 10^6^ EID_50_/0.1 ml inoculated group at 8 h post-inoculation (hpi) to prevent direct exposure to residues of the initial inoculum. Oropharyngeal (OP) and cloacal (CL) swabs were collected from 10^6^ EID_50_ inoculated and contact birds at 1, 2, 3, 4, 5, 6, 7, 10, and 14 dpi. At 14 dpi, serum was collected from surviving birds and tested for antibodies by cELISA and hemagglutination inhibition (HI) assay using homologous antigens, in accordance with WOAH guidelines. A bird was considered infected if it was either positive for virus shed at any time or had detectable antibodies at 14 dpi.

To assess viral replication in tissues, three chickens and three ducks per virus were inoculated intranasally with 10^6^ EID_50_/0.1 ml and euthanized at 3 dpi for virus titration of internal organs. At necropsy, 12 tissues (trachea, thymus, heart, lung, kidney, brain, pancreas, cecal tonsil, liver, spleen, muscle, and proventriculus) were collected. Swab samples and tissue homogenates (0.1 g/ml) were inoculated into DF-1 cells, a cell line derived from chicken embryo fibroblasts. Viral titers were expressed as log_10_ TCID_50_/0.1 ml and calculated using the Reed–Muench method. The samples with tiers below the limit of detection were assigned a value of 1 log_10_ TCID_50_/0.1 ml. To identify a significant difference in experimental data, viral titers were analyzed using an unpaired Student's t-test by GraphPad software, version 8 (GraphPad Software Inc., SanDiego, CA, USA). A *p*-values less than 0.05 (*p* < 0.05) were considered statistically significant. All animal experiments were approved by the Institutional Animal Care and Use Committee of the Animal and Plant Quarantine Agency (APQA; approval nos. 2023-765 and 2024-1458).

## Results

In SPF chickens, both viruses were identified as HPAI according to the WOAH standard, with IVPI values of 3.0 for D449(H5N6) and 2.98 for WF369-1(H5N3). Chickens inoculated with 10^6^ EID_50_ of D449(H5N6) developed clinical signs including depression and greenish diarrhea at 1–4 dpi, resulting in 100% mortality, a mean death time (MDT) of 2.6 days, and a cLD_50_ of 10^4.5^ EID_50_. Similarly, all chickens inoculated with 10^6^ EID_50_ of WF369-1(H5N3) died within 3 dpi and exhibited depression, greenish diarrhea, and cyanosis. Among contact chickens, D449(H5N6) resulted in 100% transmission and mortality, with an MDT of 7 days. In contrast, no transmission was observed in chickens exposed to WF369-1(H5N3; Table 1 and Supplementary Figure S1). Seroconversion was not detected in surviving chickens.

**Table 1:** Pathogenicity and transmissibility of D449(H5N6) and WF369-1(H5N3) in SPF chickens and ducks.

Birds species	Virus	IVPI	Dose (EID_50_/ 0.1 ml)	Mortality (%)	MDT	HI titer^a^ (log2, mean ±SD; positive no.)	cELISA^a^ (positive no.)	cLD_50_ (EID_50_/ 0.1 ml)	${\text{bid}}_{50}^{\text{b}}$ (EID_50_/ 0.1 ml)
Chicken	D449 (H5N6)	3.0	10^6^	5/5 (100)	2.6	NT	NT	4.5	—
			10^5^	5/5 (100)	3.4	NT	NT		
			10^4^	0/5 (0)	—	(0/5)	0/5		
			10^3^	0/5 (0)	—	–(0/5)	0/5		
			Contact	3/3 (100)	7	NT	NT		
	WF369-1 (H5N3)	2.98	10^6^	5/5 (100)	2.2	NT	NT	4.5	—
			10^5^	4/5 (80)	2.75	–(0/1)	0/1		
			10^4^	1/5 (20)	2.5	–(0/4)	0/4		
			10^3^	0/5 (0)	—	–(0/5)	0/5		
			Contact	0/3 (0)	—	–(0/3)	0/3		
Duck	D449 (H5N6)	—	10^6^	1/5 (20)	8	8 (4/4)	4/4	—	< 2
			10^4^	0/5 (0)	—	6.8 ± 0.4 (5/5)	5/5		
			10^2^	0/5 (0)	—	7.2 ± 0.4 (5/5)	5/5		
			Contact	0/3 (0)	—	7 ± 1 (3/3)	3/3		
	WF369-1 (H5N3)	—	10^6^	2/5 (40)	6	3.5 ± 0.5 (2/3)	3/3	—	5
			10^4^	0/5 (0)	—	–(0/5)	0/5		
			10^2^	0/5 (0)	—	–(0/5)	0/5		
			Contact	1/3 (33)	8	3 (2/2)	2/2		

^a^Seroconversion was confirmed by HI assay and cELISA with surviving birds at 14 days post-infection.

^b^An infection determined by a positive serologic response.

SPF chickens and ducks were inoculated intranasally with serial dilutions of D449(H5N6) and WF369-1(H5N3).

IVPI, intravenous pathogenicity index; cLD_50_, chicken lethal dose; MDT, mean death time; NT, not tested; BID50, bird infectious dose.

**Figure 1:**
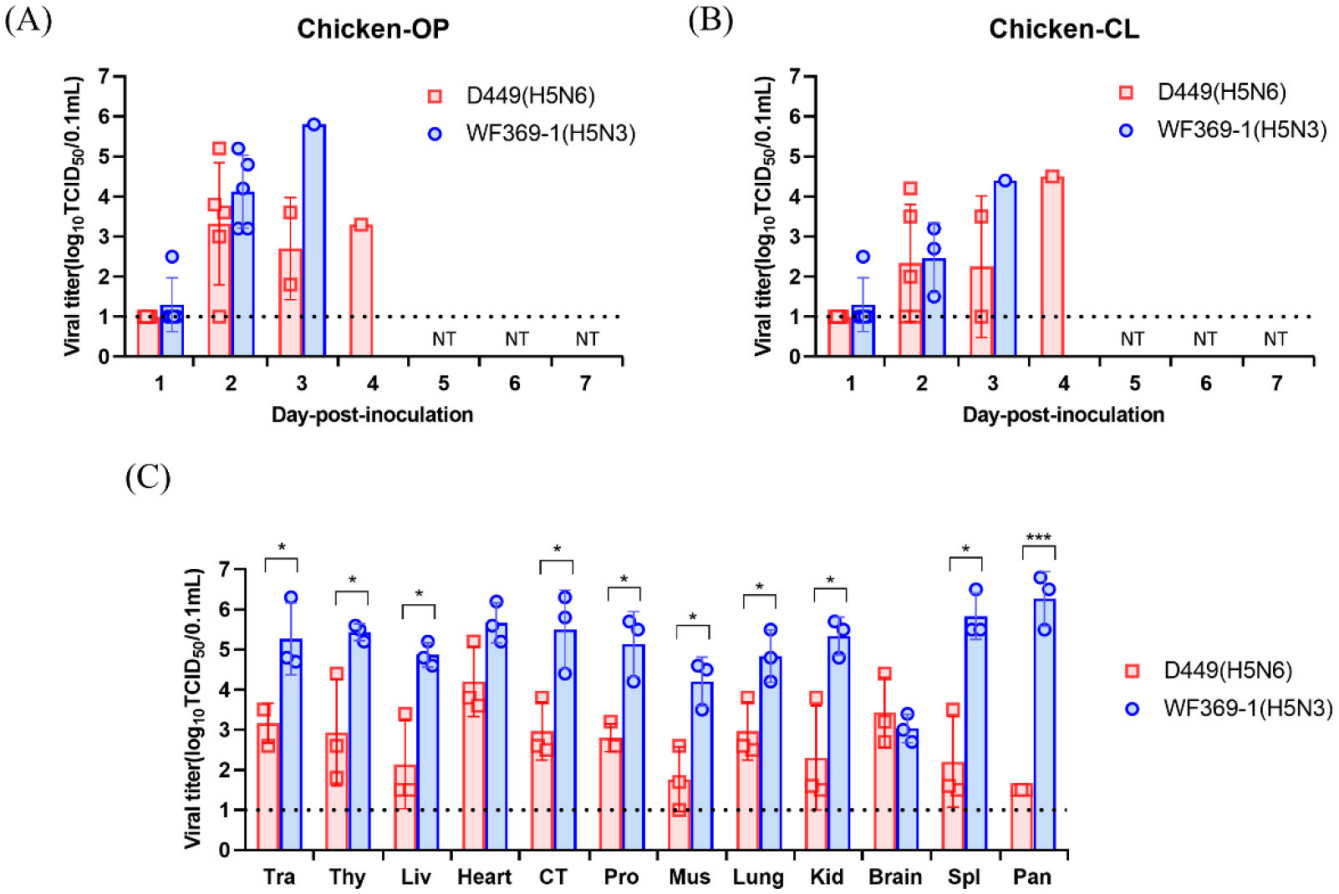
Virus titers in swabs and organs of SPF chickens inoculated with D449(H5N6) or WF369-1(H5N3). Five specific pathogen–free (SPF) chickens were inoculated intranasally with 10^6^ EID_50_/0.1 ml of each virus. Virus isolation from oropharyngeal (OP) and cloacal (CL) swab samples of chickens inoculated with **(A)** D449(H5N6) or **(B)** WF369-1(H5N3) is shown. **(C)** Organ tissues were collected at 3 dpi and homogenized to 10% (w/v), and viral titers were determined in chicken embryo fibroblast (DF-1) cells. Viral titers are presented as mean ± standard deviation. Limit of detection is 1 log_10_TCID_50_. NT, not tested due to 100% mortality; *p < 0.05, ***p < 0.0005.

To assess viral shedding, OP and CL swabs were collected from chickens inoculated with 10^6^ EID_50_ of each virus. In the D449(H5N6) group, viral shedding was detected at 1–4 dpi, with peak titers at 2 dpi in OP swabs (10^3.0^-10^5.2^ TCID_50_/0.1 ml) and at 4 dpi in CL swabs (10^4.5^ TCID_50_/0.1 ml; Figures 1A, B). In chickens inoculated with WF369-1(H5N3), viral shedding was observed at 1–3 dpi, with peak titers at 3 dpi in both OP (10^5.8^ TCID_50_/0.1 ml) and CL swabs (10^4.4^ TCID_50_/0.1 ml; Figures 1A, B).

At 3 dpi, viral replication was detected in all examined organs of chickens infected with D449(H5N6) and WF369-1(H5N3), with titers ranging from 10^1.5^ to 10^4.2^ TCID_50_/0.1 ml and from 10^3.0^ to 10^6.2^ TCID_50_/0.1ml, respectively (Figure 1C). Although both viruses were detected in all tissues, WF369-1(H5N3) were significantly higher titers than D449(H5N6) in the trachea, thymus, liver, cecal tonsil, proventriculus, muscle, lung, kidney, spleen (*p* < 0.05), and pancreas (*p* < 0.0005).

Ducks inoculated with D449(H5N6) exhibited respiratory signs such as nasal snicking sounds, greenish diarrhea, fever, depression, ocular swelling, and oral mucosa inflammation at 4–7 dpi. Mortality was 20%, with an MDT of 8 days. The BID_50_ was < 2 log_10_ EID_50_, based on seroconversion, as 100% of ducks in all dose groups were positive by HI assay and cELISA (Table 1). In contrast, ducks inoculated with WF369-1(H5N3) showed neurological symptoms, including head tremors, immobility, and corneal opacity at 5–8 dpi. Two ducks with neurologic signs died after 6 dpi, resulting in 40% mortality and a BID_50_ of 5 log_10_ EID_50_. In the contact groups, seroconversion was confirmed in surviving ducks at 14 dpi, with no mortality in the D449(H5N6) group and 33% mortality in the WF369-1(H5N3) group (Table 1). Notably, all ducks in D449(H5N6) group exhibited respiratory signs. In contrast, one duck in WF369(H5N3) group presented with neurologic signs at 7 dpi, and subsequently died, while the surviving ducks remained asymptomatic.

**Figure 2:**
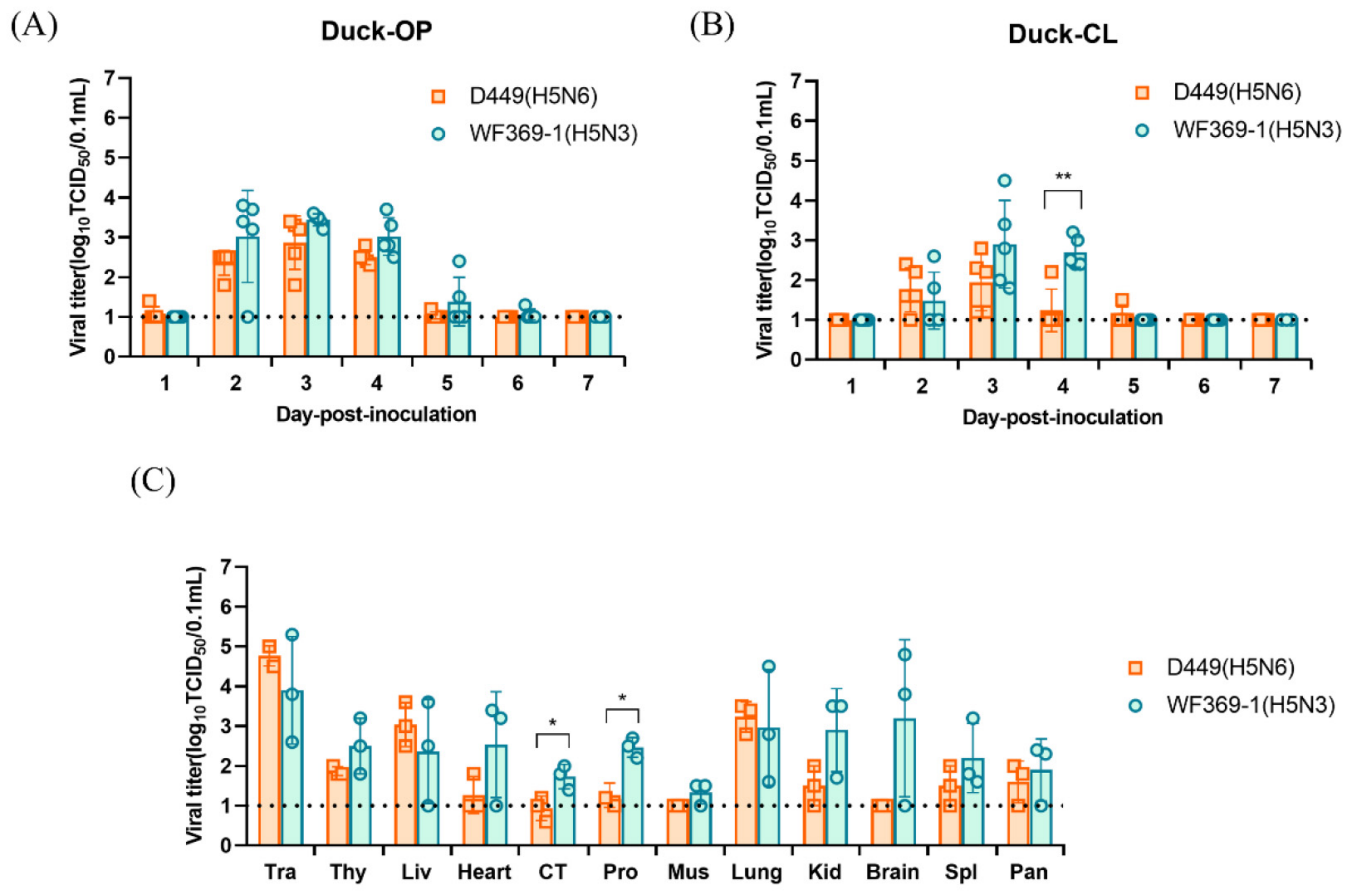
Virus titers in swabs and organs of ducks inoculated with D449(H5N6) or WF369-1(H5N3). Five ducks were inoculated intranasally with 10^6^ EID_50_/0.1 ml of each virus. Virus isolation from oropharyngeal (OP) and cloacal (CL) swab samples of ducks inoculated with **(A)** D449(H5N6) or **(B)** WF369-1(H5N3) is shown. Viral titers are presented as mean ± standard deviation. **(C)** Organ tissues were collected at 3 dpi and homogenized to 10% (w/v), and viral titers were determined in chicken embryo fibroblast (DF-1) cells. Data represent the mean of virus-positive organs with quantifiable titers. *p < 0.05, **p < 0.005.

Viral shedding in ducks inoculated with D449(H5N6) was detected in both OP and CL swabs for up to 6 dpi, with peak titers observed at 3 dpi (10^1.8^-10^3.4^ TCID_50_/0.1 ml in OP and 10^1.2^-10^2.8^ TCID_50_/0.1 ml in CL; Figures 2A, B). Ducks infected with WF369-1(H5N3) also exhibited peak shedding at 3 dpi, with titers of 10^3.2^-10^3.5^ TCID_50_/0.1 ml in OP and 10^1.8^-10^4.5^ TCID_50_/0.1 ml in CL, which were higher overall than those of the ducks infected with D449(H5N6; Figures 2A, B). At 3 dpi, D449(H5N6) was detected in most organs, except the muscle and brain, with titers ranging from 10^1.2^ to 10^3.9^ TCID_50_/0.1 ml (Figure 2C). The highest titer was observed in the trachea (10^3.9^ TCID_50_/0.1 ml). In contrast, WF369-1(H5N3) was replicated in all examined organs, with titers ranging from 10^0.8^ to 10^4.7^ TCID_50_/0.1 ml. Viral titers in WF369-1(H5N3) infected ducks were peaked at 10^4.3^ TCID_50_/0.1 ml in the brain, and significantly higher compared to D449(H5N6) in cecal tonsil, proventriculus (*p* < 0.05).

## Discussion

We assessed the pathogenicity and transmissibility of two H5 HPAIV subtypes isolated in South Korea in chickens and ducks. D449(H5N6) and WF369-1(H5N3) caused dose-dependent mortality in SPF chickens, with identical cLD50 values of 10^4.5^ EID_50_, indicating that comparable infectious doses were required to cause mortality. The results for D449(H5N6) in chickens are consistent with previous reports of Korean H5 HPAIVs, which demonstrated 100% mortality within 5 days and high transmissibility (13, 17). Similarly, a Japanese H5N6 isolate with an identical gene constellation induced complete mortality in chickens, with an MDT of 2.3 days and a cLD50 of 10^4.6^ EID_50_ (9).

Viral transmissibility is influenced by receptor-binding affinity, host factors, and environmental conditions (18–20). Although WF369-1(H5N3) exhibited high viral shedding and substantial viral loads in multiple organs of inoculated chickens, it showed no transmission to contact chickens under experimental conditions. This limited transmissibility was likely attributable to short exposure period associated with a rapid MDT of 2.2 days (Table 1) (21–23). Since viral shedding in contact chickens is typically detectable after 3 dpi, the brief infectious period likely reduced opportunities for environmental exposure (13, 14, 17, 24). Nevertheless, controlled experimental settings differ from field conditions, where exposure durations may be prolonged and biosecurity practices variable such as bird density and environmental persistence on a farm. Furthermore, receptor binding affinity may have contributed to the lack of horizontal transmission. Although clade 2.3.4.4b H5N3 HPAIV has been detected infrequently in wild birds, its potential introduction and spread within poultry populations cannot be excluded under field conditions, given the more variable and unpredictable exposure dynamics.

Respiratory signs, including labored breathing and dyspnea, are uncommon in ducks infected with H5N1 HPAIVs and have been correlated with elevated viral titers in the lungs and trachea (25, 26). Ducks infected with D449(H5N6) exhibited atypical respiratory manifestations, characterized by nasal snicking sounds, possibly resulting from partial airway obstruction. Consistent with these observations, viral titers were highest in the trachea and lungs, suggesting that the respiratory signs were associated with enhanced viral replication in the respiratory tract. Tracheal congestion has also been reported frequently during the necropsy of duck carcasses collected from H5N6 HPAI-positive farms in South Korea (27). Ducks exposed to D449(H5N6) showed high infectivity and efficient transmission with a high seroconversion rate, supporting a robust humoral immune response. This H5N6 HPAIV contains an 11-amino acid (positions 58–68) deletion in the stalk region of the NA protein, which is associated with loss of glycosylation and enhanced virulence in chickens (28, 29). In previous studies, chicken-adapted viruses with shorten NA retained the respiratory-tropic replication pattern in ducks (30, 31). Truncation or deglycosylation of the NA stalk enhanced the virulence and viral replication, resulting the increased potential for horizontal transmission in ducks (39). Further studies are needed to better understand these pathogenic characteristics.

Ducks infected with WF369-1(H5N3) exhibited peak viral replication in the brain, accompanied by severe neurological signs. In contrast, ducks infected with D449(H5N6) showed no neurological manifestations, consistent with the absence of detectable viral replication in brain tissue. These neurological symptoms have been widely reported in ducks infected with clade 2.3.4.4b H5 HPAIVs, and are associated with early invasion of the central nervous system resulting from pre-dominant neurotropism of HPAIVs in ducks (32, 33). The H5 HPAIVs with genetically diverse gene constellations have been associated with neurological symptoms in ducks, although the mechanisms underlying these manifestations remain unclear (33–36). Our findings indicate that H5 HPAIVs differ in neurotropism and this variation reflects strain-specific replication patterns and pathogenic mechanisms influenced by viral genotype, host species, and host age (37, 38).

In the present study, both the H5N3 and H5N6 viruses were highly pathogenic; however, infection outcomes differed between strains in chickens and ducks. Notably, infected ducks exhibited distinct clinical manifestations, such as respiratory and neurological symptoms. These strain-specific differences in virulence and transmissibility provide relevant information for early detection of viruses and disease control strategies in poultry. To improve understanding of the field situation, continuous pathogenicity assessments are required, while integrating experimental infection studies with complementary alternative methods may reduce reliance on *in vivo* models. Our findings underscore the need for continued investigation to offer additional insight for field-level risk assessments.

## Data Availability

The original contributions presented in the study are included in the article/Supplementary material, further inquiries can be directed to the corresponding author.

## Supplementary material

Supplementary Figure S1 Surviving rate of chickens **(A)** and ducks **(B)** inoculated with D449(H5N6) or WF369-1(H5N3), including the contact group. Birds (*n* = 5 per group) were inoculated intranasally with serial dilutions 10^2 − 6^ EID_50_/0.1 ml of each virus. Contact birds were added to isolators at 8 h post-inoculation.

Supplementary Table S1 Clade 2.3.4.4b H5N6 and H5N3 HPAIVs.
