# Supplementary material for: Pathogenicity in chickens and ducks of clade 2.3.4.4b H5N6 and H5N3 high pathogenicity avian influenza viruses in South Korea
Source: Front Vet Sci. 2026 Jun 26;13:1833958. doi: 10.3389/fvets.2026.1833958 (PMC13349917; doi:10.3389/fvets.2026.1833958)
Supplement: Supplementary Figure S1 — Surviving rate of chickens (A) and ducks (B) inoculated with D449(H5N6) or WF369-1(H5N3), including the contact group. Birds (n = 5 per group) were inoculated intranasally with serial dilutions 102 − 6 EID50/0.1 ml of each virus. Contact birds were added to isolators at 8 h post-inoculation. [file Data_Sheet_1.PDF]

Supplement table S1. Clade 2.3.4.4b H5N6 and H5N3 HPAIVs

Virus name	Collection date	Host		Genotype*	Sample type	Region	latitude	longitude	GISIAD accession no	Genotype*
A/duck/Korea/D449/2023(H5N6)	2023-12-05	Poultry	Duck	23G2	Swab	Jeonnam	34°52'11"	126°32'2"	EPI_ISL_19884235	23G2
A/Northern pintail/Korea/WF369-1/2024(H5N3)	2024-10-02	Wild bird	Northen pintail	H5N3(24G0+N3)	Feces	Jeonbuk	35°52'18"	126°41'9"	EPI_ISL_19884294	H5N3(24G0+N3)

* The details on the genotypes are previously described in a published paper (Cha et al., 2026)

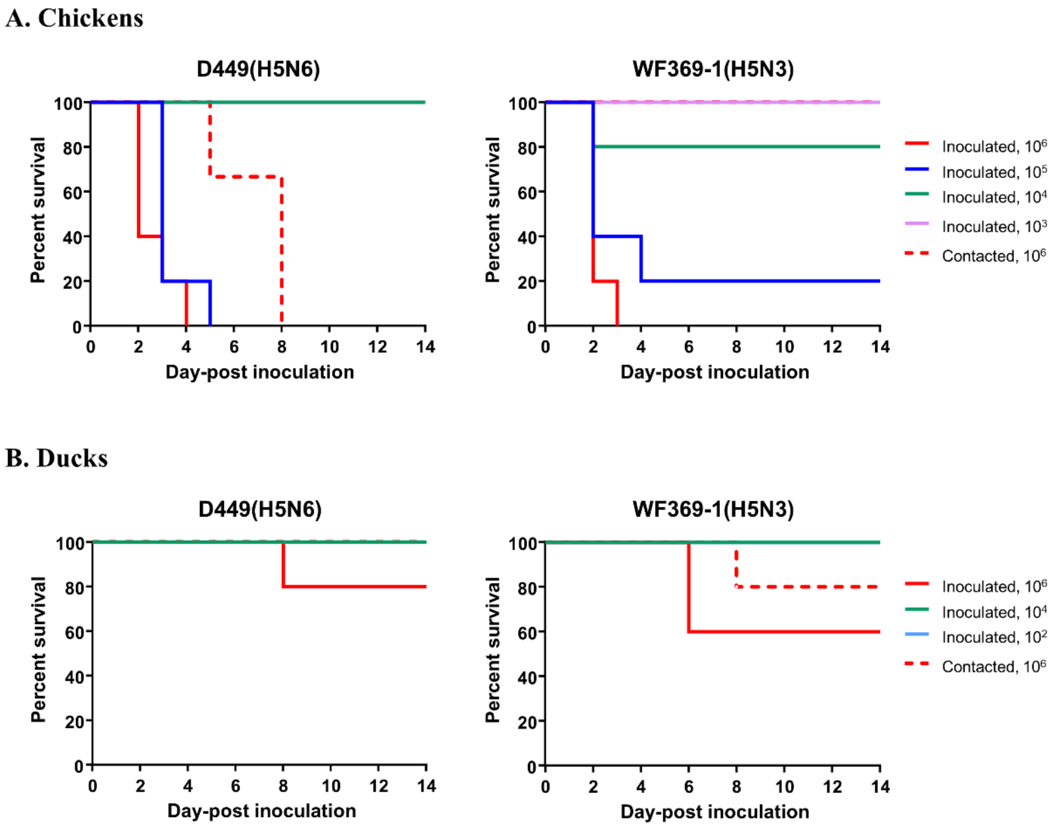

Supplement figure S1. Surviving rate of chickens (A) and ducks (B) inoculated with D449(H5N6) or WF369-1(H5N3), including the contact group. Birds (n = 5 per group) were inoculated intranasally with serial dilutions $10^{2\text{-}6}$ EID_{50}/0.1 mL of each virus. Contact birds were added to isolators at 8 h post-inoculation
